# Stress and Emotional Intelligence Shape Giving Behavior: Are There Different Effects of Social, Cognitive, and Emotional Stress?

**DOI:** 10.3389/fpsyg.2022.800742

**Published:** 2022-02-24

**Authors:** Ani Hovnanyan, Libera Ylenia Mastromatteo, Enrico Rubaltelli, Sara Scrimin

**Affiliations:** Department of Developmental Psychology and Socialization, University of Padova, Padua, Italy

**Keywords:** acute stress, prosocial behavior, willingness to help, donation behavior, trait emotional intelligence

## Abstract

Acute stress has been linked with prosocial behavior, yet it is entirely unexplored how different types of stressors may affect individuals’ willingness to help: This is particularly relevant while people is experiencing multiple sources of stress due to the COVID-19 pandemic. Here we explore whether different types of stress influence peoples’ giving behavior and the moderating role of emotional intelligence (EI). Undergraduate students were exposed to experimentally induced social, cognitive, or emotional stress and were asked to self-report on their willingness to help and donate to a charity raising funds for COVID-19 and flu patients. Results showed that when compared to a control condition, after being exposed to a social stress, participants were more willing to help a person in need. Our results also provide evidence that, after experiencing a social stress, participants with high (vs low) trait EI were more willing to help, and, as a result, donated more. Findings indicate that moderate levels of distress are associated with increased donations. Interestingly, when stress is not too threatening, high EI can regulate it and promote prosocial behaviors.

## Introduction

Since ancient times, philosophers have considered the act of one person helping another as the greatest of human values. Indeed, prosocial acts are fundamental features of a healthy and well-functioning society (Nelson et al., 2016; Van Tongeren et al., 2016). It is unquestionable that humans are prosocial species willing to help others. Prosocial behavior is defined as individuals’ voluntary intention to serve others at a temporary cost to the self (Eisenberg and Miller, 1987). However, such behaviors are influenced by situational factors (Berger and Rodkin, 2012) as well as how we emotionally respond to them (Lerner and Keltner, 2000). Individuals are constantly exposed to internal demands and environmental sources of stress, that is events that are perceived to be threatening to the self and well-being, which may influence how willing they are to help others.

The possible relation between stress and prosocial behavior has been poorly studied (Von Dawans et al., 2012), for example data are lacking on the effects of different types of stress on willingness to help and donate. Yet, this information is particularly important while humanity is facing a major worldwide health emergency.

Several types of stress fill our daily life and significantly differ one another in terms of how individuals perceive and respond to them. This in turn shapes our behaviors (Starcke and Brand, 2012) including whether we are willing or not to help others (Youssef et al., 2012). A large body of work has investigated the factors that influence willingness to help others (Agnoli et al., 2015), however, the role played by different types of stress has not been studied systematically. Thus, one of the aims of this study is to assess how willingness to help changes depending on the types of stress individuals encounter and the affective reactions experienced in response to them.

Furthermore, stress responses vary significantly among individuals in relation to how effectively they regulate their emotions. The impact of stress on willingness to help has also been found to be moderated by emotional intelligence (Agnoli et al., 2015).

The goal of the present work is to clarify the relationship between different types of stress and emotional intelligence in shaping willingness to help, while considering the exceptional condition imposed by the COVID-19 pandemic.^1^

Stress can be defined as an adaptive way to mobilize energy and motivate behavior when facing danger (Sapolsky et al., 2000; McEwen and Akil, 2020) as such we here conceptualize responses to stress in terms of a set of mechanisms (biological and behavioral) that enhance survival and that are mediated by dispositional factors within the individual (Kim and Diamond, 2002). When an individual faces a source of stress a complex set of neurohormonal response will take place together with a general unspecific physiological response that can also be linked with a specific subjective emotional experience (Del Giudice et al., 2018). This complex set of responses are thought to fluctuate based upon the intensity, nature, and duration of the stressor, as well as several internal factors of the individual experiencing it (Joëls and Baram, 2009). The way individuals respond to stressful events is determined by one’s perception of the event that can be both unconscious (Porges, 2007) and conscious (Lazarus and Folkman, 1984). In addition, the effects of stress on the functioning of the individual follows a non-linear trend: moderate stress and arousal are often adaptive and can bolster performance, whereas high levels of stress sometimes impair behavioral performance (Yerkes and Dodson, 1908; Sandi, 2013). Some studies have shown that, several factors moderate this relationship. One of these factors is thought to be the type of task, for example tasks that are more cognitively demanding require grater arousal for a better performance (Sandi, 2013). Last, different types of stress may trigger different affective, cognitive, and behavioral responses. Affective response to different stressors influences individuals’ appraisal of the environment or situation, which can lead to different choices or decisions, for example oriented toward or away from others (Lerner and Keltner, 2000).

Recent work in the field of prosocial behavior and charitable giving has shown the central role played by affect heuristic (Slovic et al., 2007). This heuristic affirms that when people make decisions they rely on their affective state (Slovic et al., 2007). So, decisions to help are significantly influenced by contextual factors (e.g., the charity people are asked to support) and people’s affective state (e.g., whether they are in a positive or negative mood; Loewenstein and Lerner, 2003). In everyday life, decisions to help others or not are frequently made under stress and this is particularly true during a worldwide sanitary emergency (Mazza et al., 2020). Given the affective response to different stressors may vary it is expected that the prosocial decisions and actions may be partly influenced by the specific affective state induced by each type of stressors. Despite the attempts to study the link between stress exposure and prosocial behavior, in terms of decision to help and donate, data are often conflicting, and several questions remain unanswered. A growing body of literature reports a positive link between exposure to stressful events and prosocial behaviors (Taylor et al., 2000; Wolf et al., 2015); however, there are also data showing a reduction in helping when people are under stress (Vinkers et al., 2013), and the effect of stress, provoked by time pressure or cognitive load, on altruistic behavior was reported to be barely significant (Tinghög et al., 2016; Fromell et al., 2020). In addition, the types of stress (e.g., social, cognitive, and emotional) and the degree experienced (from low to high) can vary significantly and, consequently, may plausibly influence prosocial behavior in specific ways.

Different situations or events may induce stress. For example, social evaluation and social exclusion (Kogler et al., 2015) or cognitive stress derived from workload and demanding tasks (Roesch et al., 2002) as well as exposure to emotional cues or situations that evoke negative and stressful emotions (van Stegeren et al., 2008). Each of these types of stress influences on one’s affective state at different levels and challenges the individual in a different way that implies the need to actively respond to restore homeostasis. In the case of a social stress, we may respond through an increased arousal and anxiety when the interaction with others seams to threaten us (Dickerson et al., 2008). Cognitive types of stress can occur when environmental demands are perceived as taxing or potentially exceeding one’s own capacity or resources to manage them, such as in complex arithmetic task when a great amount of cognitive effort needs to be used to solve the problem (Van Bockstaele et al., 2020). Emotional stress is linked with the exposure to highly negative events, cues or even thoughts that cause strong emotional distress and the mobilization of a significant amount of energy to deal with the triggered negative emotions (Mendelson, 2013).

Social, cognitive, and emotional types of stress generate the mobilization of resources that are needed to restore homeostasis; such resources might be linked to different behaviors aimed toward or away from others partly depending on the level of stress experienced (Wolf et al., 2015). In other words, the way an individual respond to a specific source of stress, and how this stress is processed by the mind and body of the individual (see the concept of neuroception proposed by Porges, 2007) may require different amount of energy in order to restore the pre-stressor balance and the selection of different behavioral responses based on a more or less conscious appraisal of the situation. Previous work on the effect of acute stress on willingness to help and donate partially backs our reasoning since, for example some evidence exists about the effect of social and cognitive stress on prosocial behavior (Sollberger et al., 2016; Tomova et al., 2017). For instance, social stress increases the frequency of donation to environmental causes (Sollberger et al., 2016) and Wolf et al. (2015) found that being exposed to social stress (TSST) enhanced emotional empathy. Additionally, there is work showing that cognitive stress increases empathy toward others in pain (Tomova et al., 2017). However, there are scant data on the effect of a purely emotional type of stress on prosocial behavior and to our knowledge there is no data simultaneously exploring the effect of different types of stress on willingness to help and donation behaviors. As a result, one of the goals of the present work is to provide evidence for the effect of emotional stress on willingness to help and donate, while, at the same time, comparing this type of stressor with those that have already been linked to prosocial behavior. Addressing this issue might give practitioners valuable information to select the best contexts in which to maximize people’s contributions.

Large variability exists in how an individual reacts to stressors as well as how the same person reacts to different stressors since the response depends on one’s appraisal of the specific situation (Lazarus and Folkman, 1984). Extensive recent work has focused on how individual differences impact people’s response to challenging or even stressful events.

One of the constructs used to assess these individual differences is trait emotional intelligence. This construct is defined as “perceived emotional self-efficacy” and measures people’s tendency to perceive and manage their emotions (Sevdalis et al., 2007). Trait EI includes a series of emotion-related personality traits and is considered as a broad and general dimension of personality (Petrides et al., 2007). Critically, Peña-Sarrionandia et al. (2015) suggested that, compared to the study of specific regulatory strategies, trait EI is a better measure of individual differences in emotion regulation. This is a key insight for our work, since the high variability in people’s responses to stress means that targeting specific regulation strategies may expose us to the risk of not capturing it. Instead, measuring trait EI we can focus on the flexibility and adaptability of people’s regulation. Consistently, Peña-Sarrionarndia and colleagues showed that people with high (vs. low) trait EI are more likely to downregulate intense emotions (such as fear, anger, or sadness) in stressful situations, and are more prone to perceive events as less negative. In line with this conclusion, Mikolajczak and Luminet (2008) have found that individuals with high trait EI appraise a stressful situation as a challenge, rather than a threat. Additionally, EI has been associated to the efficient processing of positive and negative emotions (Fernández-Berrocal and Extremera, 2006). So, it is possible that individuals with high (vs low) EI have faster mood recovery after being exposed to negative or stressful events (Salovey et al., 2002). Finally, existing data report that people with high (vs. low) trait EI tend to be more effective at stress management and to have superior levels of trait happiness, trait optimism, and self-esteem (Petrides, 2009). For instance, people with high trait EI report lower levels of occupational or life stress than their low EI counterparts (Mikolajczak et al., 2006). To our knowledge, the moderating effect of trait EI on the relationship between stress and prosocial behavior has seldom be tested, especially when looking at different types of stressors. There is a lack of understanding on how EI may affect prosocial behavior in terms of individuals’ willingness to help and donate when experiencing stress.

The goal of the present study is to assess the relationship between different types of acute stress and willingness to help and donating behaviors also considering the role of emotional intelligence. In addition, given that data were collected during the 2020 COVID-19 pandemic, we also considered whether willingness to help and donations change as a function of the target of the donation. We assessed whether participants were more willing to give to a charity collecting funds for either COVID-19 or flu patients and their families. To achieve this goal, we designed a 4 × 2 experiment in which participants were randomly exposed to one of the four stress/control conditions (e.g., cognitive, social, emotional stress or control condition), while all were presented with the two charity scenarios.

Specifically, we aimed at answering the following research questions (RQ).

RQ1a) Does willingness to help change as a function of the type of stress experienced by participants (i.e., cognitive, emotional, and social stress vs. control)?

RQ1b) Furthermore, does willingness to help change as a function of the target of the donation (i.e., COVID-19 vs. flu)?

Given the previously reported relationship between social and cognitive stress and willingness to help (Sollberger et al., 2016; Tomova et al., 2017), it is expected a positive change in willingness to help after the exposure to those types of stress. While for the effect of emotional stress on willingness to help remains to be explored. It is hypothesized that people will be more willing to help COVID-19 (vs flu) patients considering their potential sensitivity to current pandemic related situation (Jones et al., 2020).

RQ2) Does people’s trait emotional intelligence moderate their willingness to help as a function of type of stress and target of the donation?

It is hypothesized that individuals with higher (vs lower) trait EI scores will be more willing to help others when exposed to stress (Agnoli et al., 2015), and that trait EI can have a moderating role on the stress and willingness to help link. In relation to whether this moderating role changes as a function of the type of stress and target of the donation, given the lack of data, no specific hypothesis can be advanced, hence this question remains exploratory.

RQ3) Does willingness to help mediate the effect of the independent variables on the actual donation behavior displayed by participants?

This research question is consistent with existing work in the domain of charitable giving showing that people’s willingness to help has an impact on their actual decision to donate (Caserotti et al., 2019). Since we expect to find that specific types of stress should have different impact on both willingness of help and donations, we should be able to find the mentioned mediation effect. Furthermore, we will also assess whether the trait EI will have a moderating role in the mediation model. As we reported above, no specific hypothesis can be advanced, and we assess the role of trait EI in an exploratory way.

## Materials and Methods

### Participants

The sample was composed of 400 undergraduate students, 200 male participants (50%) with a mean age of 24.2 (*SD* = 4.72). Each of four conditions comprised 100 students balanced for gender (50/50). Students of developmental psychology course, at the University of Padova, were invited to participate in the study in exchange for course credits.

### Procedure

**FIGURE 1:**
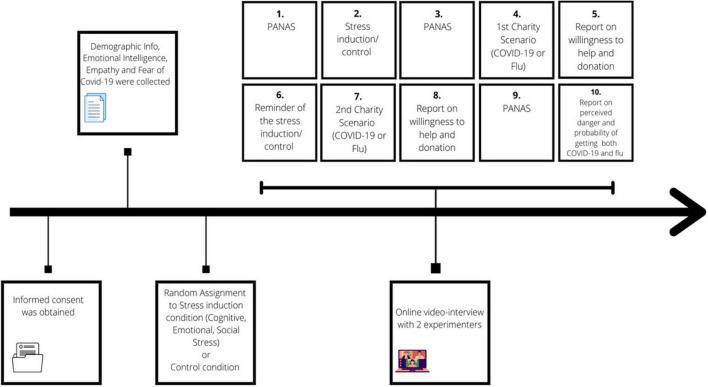
Graphical representation of study procedure. The overall data collection lasted approximately 3 months, containing three different sessions and the distance between one and the other session was kept similar per each participant. The first session included the study procedure description when the informed consent was obtained as well. From 2 to 3 days later, they were sent an online questionnaire on demographic data and individual variables lasting approximately 10 min. One week later, after being randomly assigned to one of the conditions, participants were invited to take part in the online video-interview lasting about 15 min. In average 5–7 interviews have been done per day for 3 months.

Data were collected on-line between October and December 2020. As shown in Figure 1, after obtaining informed consent from participants, an initial survey allowed to collect demographic information together with data on fear of COVID-19, trait emotional intelligence and empathy. Subsequently, participants were invited to take part in an online video-interview with two experimenters to investigate the effect of stress on willingness to help and donate. Participants filled in the initial questionnaires at the beginning of the data collection and scheduled their call in within one week after they provided the first information. We did approximately 5–7 interviews per day. The rational for the video-call was to assure that the participants remained focused on the task and did not avoid the stress exposure. Overall, five experimenters were involved in the study while two experimenters for each interview were randomly assigned among conditions. During the interviews, participants were not requested to talk, but type or chose preferred answers. Participants were randomly assigned to one of the four conditions: three included the exposure to different types of stress (social; cognitive; and emotional) while one was a no-stress control condition. Before and after the stress or control task exposure, participants were asked to self-report on their negative affect. After the stress exposure, to measure willingness to help and donation behavior, all participants were presented with the description of a charity raising funds for a very ill COVID-19 or flu patient.^2^ Participants were later asked to self-report on their willingness to help the patient and the amount of money they were willing to donate to the organization. Subsequently, participants were exposed to a short reminder of the stressful/control task they had experienced before and were then asked to read the other patient scenario and self-report on willingness to help and donate. The scenarios were randomized within condition, so that 50% of the participants were exposed to COVID-19 case after the task and the flu case after the reminder and vice versa. Lastly, they were asked to self-report on perceived danger of COVID-19 and flu, and the probability of getting the viruses.

### Measures

#### Stress Induction Conditions

The Opensesame software (Mathôt et al., 2012) was used to develop online manipulations, and the duration of each condition was assured to be approximately the same (around 13 min).

##### Social Stress

To induce social stress, participants were exposed to an on-line version of the Sing-a-Song Stress Test (SSST; Brouwer and Hogervorst, 2014). In the present study the SSST was administered in an internet-based version (e-SSST), but the stimuli and overall duration of the task were comparable with the original task. Participants were requested to sit comfortably and read the phrases appearing on the monitor one of which contained a task (task essence was not specified). Nine neutral phrases with the same length were selected from Italian Wikipedia (e.g., “The body of the average human adult male is about 60–63% water and the average adult female is about 52–55%”), and were presented for 8,000 ms. The 10-th phrase contained the task: “Please, choose a random song and start singing in a loud voice. We are registering your performance so that our colleagues can watch and judge it later. Once you are ready, please, press the button and keep singing till the ‘Rec’ disappears.” The recording simulation was done with the “Rec” icon being active at the right top of the screen for 3 min (duration was not previously specified). In the end, they got a message that the registered performance will be sent for evaluation. During the reminder, they were asked to sing a short piece of song (“Rec” lasting for 1 min); this second part of the task was justified by saying that we need to make sure the recording went well.

##### Cognitive Stress

To induce cognitive stress, a mental arithmetic task was adopted, following previously used protocol (Qi et al., 2017). Six blocks (2 × 2 × 2) of addition, subtraction and multiplication expressions were presented respectively with one (e.g., 3.4 + 6.3) and two decimal numbers (e.g., 2.06 × 4.72) so that each block contained seven arithmetic expressions of the same type while the expressions containing one or two decimals presented randomly. Under time pressure, participants were asked to estimate whether the result of each calculation would be above 10 or not by pressing “z” or “m” keys. At each block, following the fixation point of 100 ms, participants were given 3,000 ms to see the calculation and to provide their response. As soon as the response was submitted (or 3,000 ms passed) the formula disappeared. After each block, participants got a feedback on their reaction time and accuracy, and in 80% of cases an automatically generated negative feedback (e.g., “Oh no, you failed, you could be faster.”) appeared despite the performance. The reminder of the task was composed by only three blocks that followed the same design.

##### Emotional Stress

To induce emotional stress, participants were exposed to 36 pictures^3^ selected from the International Affected Picture System (IAPS; Lang et al., 2008). Based on IAPS norms, all the pictures had a negative valence (2.0 or less) and with high arousal (at least 6.0) which have been reported to correspond to the ranges of pictures inducing negative stress (van Stegeren et al., 2008). Participants were asked to sit comfortably and watch the pictures, each lasting 8,000 ms and following one after another. The sequence of the stimuli was the same for all participants. The reminder of the task was composed by 16 distress inducing pictures following the same procedure.

##### Control Task

The no-stress control task was developed based on a standardized low-cognitive-demand task (Plain Vanilla; Jennings et al., 1992). Participants were asked to watch images containing gray balls of different shapes and positions at each stimulus, and to count the cases when a green rectangle appears. Thirty-six images of 8,000ms each were presented among which nine images contained a rectangle. The reminder task consisted of 12 images with three rectangle cases.

#### Charity Scenarios

To measure willingness to help and donation behavior, all participants were presented with two scenarios describing a case of a very ill COVID-19 or flu patient for whom a charitable organization was collecting funds (see Supplementary Figure 1). Specifically, participants were instructed to read an article on a serious case of a COVID-19 or flu patient. Both the COVID-19 and flu articles had the same length (one page), and structure and the patients’ pictures were balanced for participants’ gender. After reading the article, they were asked to self-report on willingness to help him/her (i.e., “If you were given a chance how much would you be willing to help him/her?”) on a scale from 0 (not at all) to 6 (very much) (adapted from CLS, Sprecher and Fehr, 2005). Lastly, participants were asked whether they were willing to donate to the charitable organization in support of the COVID-19 and flu patients and if yes how much they were willing to donate on a scale ranging from 0 to 10 euros (e.g., “Imagine having 10 euros in your wallet, would you like to donate money for this patient? If yes, how much would you donate (0–10)?”).

#### Trait Emotional Intelligence

The TEIQue-SF (Petrides, 2009) is a 30-item self-report scale that measures trait EI using a 7-point scale ranging from 1 (completely disagree) to 7 (completely agree). Items ask participants about their tendency to perceive, regulate, and express their emotions (e.g., “I usually find it difficult to regulate my emotions; I often pause and think about my feelings”). The internal reliability of the scale was high in this study (Cronbach’s α = 0.86).

#### Control Variables

##### Changes in Negative Affect

To assess the changes in negative affect before and after the stress induction procedure the Negative affect subscale of the Positive and Negative Affect Schedule (PANAS; Watson et al., 1988) was used. Specifically, we asked participants to indicate the extent to which they feel in a specific way at that moment from 0 (not at all) to 4 (extremely). The list of all the negative affective states is presented in Supplementary Table 1. The scale showed a high internal reliability in this study (Cronbach’s α = 0.90). For the analysis, we have computed the delta PANAS which is the difference between the PANAS 2 (after stress) and the PANAS 1 (before stress). It is worth noting that, having a self-report measure of how participants perceive their affective response after being exposed to different stressors might offer important information on the conscious subjective component of the specific response activated after each type of stressor.

##### Fear Related to COVID-19

The fear of COVID-19 scale (Ahorsu et al., 2020; Soraci et al., 2020) was used to measure participants’ fear of the virus. It is a 7-item self-report scale asking the participants to report on the extent to which they agree or disagree with the presented statements using a 5-point scale ranging from 1 (strongly disagree) to 5 (strongly agree) (e.g., “I am afraid of losing my life because of Corona”). The scale’s internal reliability was high in this study (Cronbach’s α = 0.87).

##### Empathy

Toronto Empathy Questionnaire (TEQ; Spreng et al., 2009) is a 16-item scale that was used to measure empathy. The internal reliability of the TEQ was high in this study (Cronbach’s α = 0.83).

## Results

### Preliminary Analyses

Descriptive statistics are presented in Table 1 (Supplementary Table 2 shows group comparisons).

**TABLE 1:** Descriptive statistics of main study variables.

		Stress type condition			
		
		Control *M*(SD)	Cognitive *M*(SD)	Emotional *M*(SD)	Social *M*(SD)
	Scenario				
Help	COVID-19	3.48(1.49)	3.60(1.60)	3.71(1.38)	3.91(1.67)
	Flu	3.30(1.42)*^a^*	3.16(1.61)*^b^*	3.54(1.34)	3.73(1.55)*^a,b^*
	COVID-19 and Flu	3.39(1.45)*^c^*	3.38(1.62)*^d^*	3.63(1.36)	3.82(1.61)*^c,d^*
Donation	COVID-19	7.55(3.23)	7.09(3.13)*^e^*	8.12(2.76)*^e^*	7.58(3.26)
	Flu	7.36(3.25)	6.63(3.33)*^f^*	7.70(2.87)*^f^*	7.26(3.18)
	COVID-19 and Flu	7.46(3.23)	6.86(3.23)*^g^*	7.91(2.82)*^g^*	7.42(3.22)
Delta PANAS		−3.64(5.63)	2.13(6.01)	9.12(9.19)	3.63(7.65)
Trait EI		4.92(0.84)	5.02(0.65)	5.02(0.70)	5.00(0.65)
Empathy		65.00(7.22)	62.02(15.38)	64.00(9.54)	62.06(13.17)
Fear of COVID-19		25.64(9.88)	24.82(8.80)	25.40(8.58)	25.74(8.31)

*Letters indicate group comparisons. ^a^t = −2.001, p = 0.046. ^b^t = −2.53, p = 0.012. ^c^t = −2.11, p = 0.035. ^d^t = −2.04, p = 0.042. ^e^t = 2.41, p = 0.016. ^f^t = 2.37, p = 0.018. ^g^t = 2.45, p = 0.014.*

Results of the correlations between main variables (Table 2) showed that willingness to help both COVID-19 and flu patients were correlated with each other and with donation behavior (both COVID-19 and flu), emotional intelligence, empathy, fear of COVID-19, age, and gender. Donation behaviors for both illnesses were correlated with each other, and with gender, while donating for COVID-19 patients was also correlated with empathy and fear of COVID-19. There was a correlation between emotional intelligence and affective state, empathy, and fear of COVID-19. And empathy was correlated with fear of COVID-19. Finally, there was a correlation between gender and almost all the variables (except the affective state); and between the age and fear of COVID-19.

**TABLE 2:** Correlation matrix between main variables.

	1	2	3	4	5	6	7	8
1. Help COVID-19								
2. Help Flu	0.72***							
3. Donation COVID-19	0.44***	0.38***						
4. Donation Flu	0.36***	0.43***	0.89***					
5. Delta PANAS	0.04	0.00	0.01	0.01				
6. Trait EI	0.11*	0.12*	0.01	0.03	0.14**			
7. Empathy	0.23***	0.17***	0.12*	0.10	0.10	0.13*		
8. Fear of COVID-19	0.24***	0.17***	0.10*	0.08	–0.05	−0.15**	0.13**	
9. Age	−0.15**	−0.11*	–0.01	0.00	–0.01	0.01	–0.04	−0.15**

*p < 0.05. **p < 0.01. ***p < 0.001.

#### Affective State

To assess whether the stress induction had an effect on participants’ affective states, we computed a delta PANAS, that is the difference between the PANAS score immediately after the stress induction and at baseline, in this way we were able to obtain an index for the change in the negative affect. Then, a multilevel linear regression was performed with type of stress (control, cognitive stress, emotional stress, and social stress) and time (baseline and after the stressor) controlling for gender. Specifically, there were significant difference between the control condition and each other type of stress across time: respectively, *B* = 1.32, *SE* = 0.57, *t* = 2.32, *p* = 0.02 for the cognitive stress, *B* = 3.94, *SE* = 0.58, *t* = 6.83, *p* < 0.001 for the emotional stress, and *B* = 1.11, *SE* = 0.57, *t* = 1.94, *p* = 0.05 for the social stress. A slope analysis showed that while in the control condition there was a significant decrease in stress over time (mean at baseline = 11.63, SD = 11.65 vs. mean at t2 = 7.99, SD = 7.90; *t* = −2.71, *p* < 0.001), a significant increase emerged after the emotional stress inductions (mean at baseline = 8.65, SD = 8.35 vs. mean at t2 = 17.77, SD = 11.19; *t* = 6.89, *p* < 0.001). No significant effect on the PANAS was found after the cognitive stress induction (mean at baseline = 11.01, SD = 10.81 vs. mean at t2 = 13.13, SD = 10.10; *t* = 0.56, *p* = 0.57) and after the social stress induction (mean at baseline = 10.32, SD = 8.46 vs. mean at t2 = 13.95, SD = 8.27; *t* = 0.04, *p* = 0.97). Hence, the change in negative affect, was included as a covariate in the following analyses. Given the important changes in the overall negative affect score, in Supplementary Table 1 we report also the changes in the single affective states composing the total score. As reported in the table, participants reported to experience high levels of “Alert,” “Ashamed” and “Nervous” states when exposed to the social stress condition (M = 0.99, SD = 1.36; M = 2.19, SD = 1.37; M = 0.68, SD = 1.32, respectively); while “Embarrassed” state was high in the cognitive stress condition (M = 0.93, SD = 1.18); and “Afraid,” “Miserable,” “Disgusted,” “Sad,” and “Shocked” were reported as high in the emotional stress condition (M = 0.63, SD = 1.13; M = 0.86, SD = 1.19; M = 2.09, SD = 1.35; M = 1.22, SD = 1.29; M = 1.55, SD = 1.36, respectively).

### Main Results

#### Stress and Willingness to Help

To assess if willingness to help changed as a function of the type of stress experienced by participants (i.e., cognitive, emotional and social stress vs. control) and in response to the target of the donation (i.e., COVID-19 vs. flu) we run a multilevel linear regression model with willingness to help as the dependent variable and type of stress and target of the donation as factors as well as a second model in which we included the interaction between type of stress and target of donation. In addition, in both models, we included as covariates: empathy, fear of COVID-19, gender, and change in negative affect. A model comparison showed that the addition of the interaction did not improve the fit to the data (*X*^2^ = 3.54, *p* = 0.32). As a result, here we discuss only the model with the main effects of type of stress and target of the donation (Table 3). We found a significant effect of target of the donation (*B* = −0.26, *SE* = 0.06, *t* = −4.48, *p* < 0.001), indicating that participants were more willing to help when the target was suffering from COVID-19 rather than flu. Furthermore, for the type of stress a significant difference emerged for the comparison between control condition and social stress (*B* = 0.53, *SE* = 0.20, *t* = 2.61, *p* = 0.01), indicating that participants were more willing to help when experiencing social stress. The differences between the control condition and the other two stress conditions were not significant (*p*s = 0.09 or higher, see also Table 1 for mean and group comparisons). Given the social stress condition was the only one different from the control, we decided to run a second analysis to assess whether any difference emerged among the three types of stress manipulations. Once we changed the reference level to the social stress condition, the results showed that it was different from the control (*B* = −0.53, *SE* = 0.20, *t* = −2.61, *p* = 0.01) and the cognitive stress conditions (*B* = −0.43, *SE* = 0.19, *t* = −2.26, *p* = 0.02), whereas the difference with the emotional stress condition was not significant (*B* = −0.14, *SE* = 0.20, *t* = −0.71, *p* = 0.48).

**TABLE 3:** Multilevel linear regression model with willingness to help as the dependent variable, type of stress and target of the donation as factors.

	*B*(SE)	*df*	*t*	*p*
(Intercept)	2.54(0.50)	402.84	5.13	0.000
Condition				
Cognitive	0.11(0.20)	378.24	0.55	0.581
Emotional	0.39(0.23)	378.12	1.71	0.088
Social	0.53(0.20)	378.18	2.61	0.009
Charity Scenarios (flu = 0; COVID-19 = 1)	0.26(0.06)	376.72	–4.48	0.000
Delta PANAS	−0.01(0.01)	389.18	–0.99	0.323
Gender	−0.00(0.00)	378.12	–3.93	0.000
Empathy	0.02(0.01)	378.46	2.69	0.007
Fear of COVID-19	0.02(0.01)	377.87	2.05	0.041

Finally, there was a significant positive effect on willingness to help for both empathy and fear of COVID-19, while females were more willing to help than males. In addition, the theoretical relevance of the covariates was also statistically supported as the model was stronger when the covariates were included (R^2^ = 0.12) compared to when they were not (R^2^ = 0.02). However, the same difference among stress manipulation on the willingness to help remained significant also when covariates were removed from the model.

#### Moderating Role of Emotional Intelligence

**FIGURE 2:**
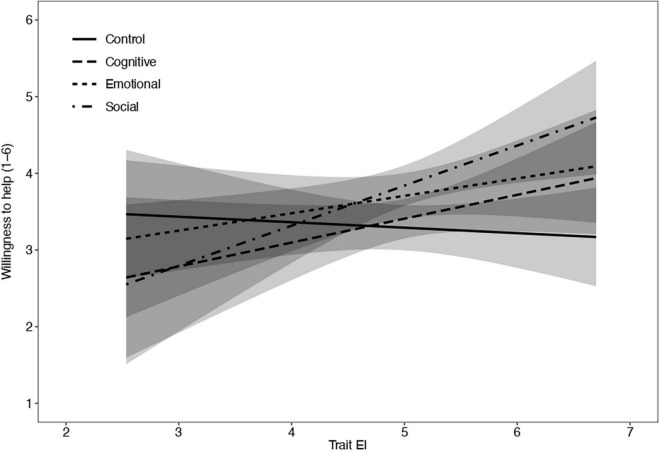
Simple slope of trait emotional intelligence predicting willingness to help for control, cognitive, emotional, and social conditions. The x-axis represents the score of trait EI, and the y-axis represents the degree of willingness to help. Conditions are represented by the types of lines.

To assess the moderating role of peoples’ trait emotional intelligence we performed a multilevel linear regression model with willingness to help as the dependent variable, type of stress and target of the donation as well as the interaction between type of stress and trait EI. In addition, we included as covariates empathy, fear of COVID-19, gender, and PANAS (Table 4). Results revealed a significant interaction between the trait EI and the contrast comparing the control condition with the social stress induction. The two contrasts including the cognitive stress induction and the emotional stress induction were not significant. All covariates that were significant in the previous analysis remained significant. A slope analysis showed that the effect of trait EI was only significant in the social stress induction condition (*t* = 2.52, *p* = 0.001) but not in all other conditions (*t*s = 1.50 or lower, *p*s = 0.14 or higher). See also Figure 2.

**TABLE 4:** Multilevel linear regression model with willingness to help as the dependent variable, type of stress and target of the donation as well as the interaction between type of stress and trait EI.

	*B*(SE)	*df*	*t*	*p*
(Intercept)	2.89(0.95)	377.07	3.04	0.002
Condition				
Control vs. Cognitive	−1.79(1.32)	367.63	–1.36	0.174
Control vs. Emotional	−1.07(1.31)	367.34	–0.82	0.411
Control vs. Social	−2.41(1.33)	367.38	–1.81	0.07
Trait EI	−0.07(0.17)	369.96	–0.43	0.667
Charity Scenarios (COVID-19, flu)	−0.26(0.06)	375.82	–4.47	0.000
Fear of COVID-19	0.02(0.01)	366.00	2.29	0.022
Gender	−0.01(0.00)	366.26	–3.67	0.000
Empathy	0.02(0.01)	366.33	2.44	0.015
Delta PANAS	−0.01(0.01)	367.91	–1.16	0.245
Condition Control vs. Cognitive × Trait EI	0.38(0.26)	367.72	1.46	0.144
Condition Control vs. Emotional × Trait EI	0.30(0.26)	367.64	1.15	0.250
Condition Control vs. Social × Trait EI	0.59(0.27)	367.51	2.24	0.025

*Baseline category for Condition was Control Condition.*

#### Donation Behavior

We then assessed participants’ donation decisions by way of a multilevel linear regression model with type of stress, target of the donation, trait EI, willingness to help, and the interaction between condition and trait EI as predictors. In addition, we included in the model the same covariates as in previous analyses. Results showed a significant effect of willingness to help (*B* = 0.93, *SE* = 0.07, *t* = 12.90, *p* < 0.001). All other effects were not significant (*p*s = 0.07 or higher).

#### Mediation Analysis

**FIGURE 3:**
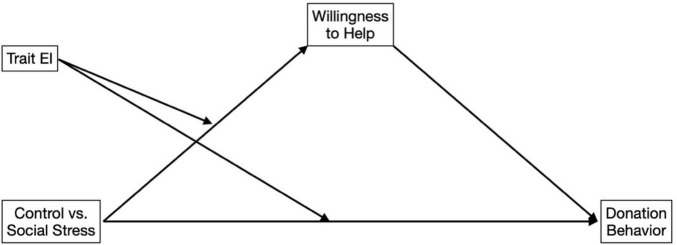
Path of the mediation analysis. The model tests whether willingness to help mediates the effect of the independent variables on donation behavior.

Lastly, we assessed whether willingness to help mediated the effect of the independent variables on the actual donation behavior displayed by participants. The tested model is presented in Figure 3. As it can be seen it included the main effects of condition (control vs. social stress induction) and trait EI as well as their interaction as predictors of both willingness to help (mediator) and donation behavior (dependent variable). We also included the same covariates as in previous analyses. Although neither the condition nor the interaction had a direct effect on donation behavior (respectively, *B* = 0.98, *SE* = 0.69, *t* = 1.43, *p* = 0.16 for condition and *B* = −0.21, *SE* = 0.14, *t* = −1.54, *p* = 0.12 for the interaction, see also Table 1 for mean and group comparisons), there was a significant indirect effect of willingness to help (*B* = 0.50, *SE* = 0.19, *t* = 2.60, *p* = 0.009). In other words, in the social stress condition compared to the control, emerged an effect of trait EI whereby an increasing score on this dimension led to an increase in willingness to help and, as a result, to higher donations.

## Discussion

This is the first study comparing the effects of social, cognitive, and emotional stressors on willingness to help and donation behavior. The aim of this study was to investigate how acute stress affects individual’s willingness to help and donation behavior when considering the potential moderating role of emotional intelligence. Given the current situation related to the COVID-19 pandemic, we assessed how willingness to help changes as a function of donation target, that is COVID-19 or flu patient.

The results are consistent with previously reported data on the negative impact of social, cognitive, and emotional stressors on people’s affective state (van Stegeren et al., 2008; Qi et al., 2017). Namely, when comparing with the control condition, all three stressors were associated with a change in negative affective state.

One of the key findings of our study is related to the comparison of the effects that different types of stress have on willingness to help. We found that participants were more willing to help after being exposed to social stress compared with the control condition. The change in negative affect participants reported in the social condition was intermediate compared to the emotional (highest) and cognitive (lowest) conditions. Based on the impact that each type of stress had on the participants, a plausible explanation of the effect of stress on willingness to help is that people are more likely to act when experiencing medium negative affect. That is, when exposed to social stress, participants were more willing to help than when exposed to the control condition. At a broader conceptual level, this data may be explained by the tend-and-befriend hypothesis proposed by Taylor et al. (2000), which assumes that at situations of stress an adaptive way to respond to stress may be the tendency to help others with the potential to have collaborative relations at future challenging conditions. While when participants were exposed to high distressing emotional images or the low distressing cognitive task no difference from the control condition was found. These findings are in line with Decety’s empathy model (Decety and Lamm, 2006) stating that other-oriented feelings and prosocial behaviors may not occur under high levels of personal distress, since it can challenge resources and activate an adaptive stress response. It is possible that for the participants who had an increased stress level after watching emotionally negative pictures, helping others would have been too demanding as they had to use their resources to manage their own reactions. Even from an evolutionary point of view, focusing on self needs at times of highly stressful situation may potentially increase one’s chances to survive. It should, however, be noted that when comparing willingness to help in the social stress condition to the other two stress conditions (i.e., emotional, and cognitive stress), no significant difference was found in participants’ willingness to help after a social and an emotional stressor. Yet, participants were more willing to help after the social stressor compared with the cognitive one. Not different effects of social and emotional stressors on willingness to help can be explained by the fact that specific elements of stress manipulations in both cases could somewhat promote prosociality, unlike at cognitive stress condition. Namely, participants knew their song would be watched and they might think their behavior would be evaluated as well, so perhaps they tried to perform “well” by their willingness to help. In the same way, stress inducing images could potentially promote helping behavior through visual cues, such as an image of a person in negative mood or in danger who might need support.

In contrast to highly stressful emotional condition, the cognitive stressor was associated with stress level not too different from those of the control condition and, thus, it did not affect participants’ willingness to help. Hence, when thinking about the relationship between stress and willingness to help we might refer to a non-linear, inverted U-shaped function as proposed by Wolf et al. (2015). That is, possibly, under low and high levels of stress individuals may be less willing to help others in need, while a medium level of stress can be associated with seeking and providing support and may lead individuals to orient toward others. Yet, previous studies linking social stress and willingness to help or more in general prosocial behavior have found that social stress exposure increased participants’ trust, trustworthiness and sharing behavior in social interaction (Von Dawans et al., 2012); as well as altruistic responses (Buchanan and Preston, 2014).

Additionally, the affective response to each stressor could have influenced the prosocial behavior as well. As such, an alternative possible explanation of why participants in the social stress condition were more willing to help could be that the affective states like alert, ashamed, and nervous that were experienced high in the social stress condition, may have potentially led to pro-social actions (compared to afraid, miserable, disgusted, sad, and shocked states that were high in emotional stress condition). More specifically, the effect of social stress induction on prosocial behavior could be due to the negative affect experienced, for instance, the participants who felt ashamed during the manipulation might want to help others to “recover” their reputation and to give a second chance to the “evaluators” to reconsider their performance.

This first analyses also showed that the participants were more willing to help when the patient was suffering because of COVID-19 (vs flu). Even though flu when data were collected was comparable with COVID-19 in terms of prevalence and mortality rates, participants demonstrated more helping intentions for COVID-19 patients. This finding may be due to the fact, that during the pandemic people are more sensitive to this specific topic and, so, give more importance to helping for COVID-19 related reasons (Jones et al., 2020). However, it is important to point out that whereas participants were more willing to help for the COVID-19 patients, the type of stress did not interact with the patient case, thus indicating that findings were not influenced by the pandemic^4^.

The second research question aimed at exploring the possible moderating effect of trait EI on the relationship between social stress and willingness to help. Results showed that participant’s willingness to help under social stress was moderated by trait EI, namely participants’ having high (vs. low) levels of EI were more willing to help under social stress. Since people with high trait EI are more effective at regulating their emotions, a possible explanation of this finding is that they were more able to regulate the negative affect elicited by the social stressor, thus being more willing to exert an effort to help others in need. This is in line with the notion that high trait EI scores may lead to efficient stress management and high trait happiness, trait optimism, self-esteem (Petrides, 2009).

Interestingly, this finding strengthens the hypothesis that a moderate level of distress can increase participants’ willingness to help, that is better self-regulatory abilities (higher trait EI) can tune down distress and promote prosocial behaviors. It should be noted here that trait EI interacts only with social stress and not with the response elicited by the emotional stress, even though exposure to this type of stressor caused a greater negative affect compared to all the other types of stress. The rational here might be that EI moderates the link between stress exposure and willingness to help when the response elicited by the stressor is associated with high arousal. We might expect that the social stress task, while eliciting less negative affect compared to the emotional stressor, caused greater arousal. This is supported by a wide literature using social types of stress such as the Trier social stress test (McRae et al., 2006) or the sing a song test (Brouwer and Hogervorst, 2014) to elicit a stress response and an increase in arousal (Eagle et al., 2021). This explanation however should be address by future studies registering the elicited response to different types of stress in particular addressing arousal, for example through registration of peripheral physiological indexes such as heart rate or skin conductance response.

Our third research question investigated whether willingness to help mediated the effect of the independent variables on the actual donation behavior displayed by participants. The mediation analysis showed that the effect of the type of stress on willingness to help led to differences in donation behavior as well. As a result, by increasing willingness to help, the social stress manipulation had the indirect effect (compared to other types of stress) of increasing how much people were willing to donate. Furthermore, participants with greater trait EI were more willing to help and, as a result, also donated more. These results could be explained by the Theory of reasoned action (TRA; Ajzen and Fishbein, 1980) and the Theory of Planned Behavior (TPB; Ajzen, 1991): both theories assume that human behavior is affected by behavioral intention. Indeed, the intention to help, expressed by participants as willingness to help, had a direct positive effect on the actual donation behavior. A possible explanation might be that when participants felt middle levels of stress, that is in the social stress condition, acting in a prosocial fashion my reduce the stress people experienced (Taylor et al., 2000; Buchanan and Preston, 2014), whereas when stress is too high or too low participants may not be able to use giving as a regulation strategy or do not need it.

Moreover, trait EI moderated the effect of social stress on willingness to help, which in turn influenced donations. Therefore, we must conclude that the indirect effect of stress on donations is not equal for all participants but depends on individual differences in emotion regulation. Indeed, on the one hand, it has been shown that there is ample variability in how specific individuals deal with stress and, on the other hand, previous work on trait EI has found it to be a good proxy of the use of more adaptive regulation strategies. As we stated in the introduction, our analysis of the role of trait EI was explorative and, as such, it should be further investigated in the future.

The present study has several limitations. Specifically, we believe that it would have been very interesting to assess the physiological correlates of stress response. We were not able to collect this data due to the pandemic, but this work would benefit from a replication study comparing the peripheral physiological responses to different types of stress and studying how it might be linked to willingness to help and donating behaviors. Moreover, the potential confounds related to the different effects of all three manipulations on prosocial behavior need to be considered. As mentioned above, the reason why participants decided to help could partly be the specific affect induced by the stressor (e.g., ashamed) and/or their belief that the experimenters may continue to evaluate their “helping performance” after singing at social stress condition. Similarly, the pictures that they were exposed to during the emotional stress condition could potentially contain visual cues (e.g., images of someone in need/danger) promoting helping behaviors. It should also be noted that the different stress manipulation tasks required acts of different nature (e.g., signing, doing arithmetic tasks, or watching images) which might somehow influence the elicited response. However, when comparing different sources of stress, it is very difficult to have the same actions involved. Once more the inclusion of physiological indexes might help to better control this issue (e.g., checking for the effect of movement, degree of sympathetic response). Overall, further investigations are needed to address all these critical aspects. Finally, the data collection was conducted online, and together with its benefits (e.g., faster communication, less financial resources) the online data collection may potentially be a source of several issues. One of the limitations of online experiments is that the experimental conditions cannot be identical for each participant, and some external factors may be uncontrollable. We made a great effort to reduce this variability to the minimum, for instance asking participants to sit alone in a quiet room, yet we may expect the stress manipulations to work better in experimental rooms specifically designed for the task rather than within home environments (it should be noted that very recent and preliminary data have shown the efficacy of on-line stress exposure in terms of emotional response (Eagle et al., 2021).

Despite the limitations, this study significantly contributes to both the literature on stress and that on willingness to help. Here we emphasize the importance of studying how specific types of stress, which potentially can be experienced at different levels, may be associated with people’s willingness to help, and donating behaviors. Findings reveal that after being exposed to social stress, which causes an intermediate (i.e., not too high, or too low) negative emotional response, people are prone to act pro-socially and help others particularly when they have high trait EI. Overall, from the present study, we can expect people to engage more in giving behaviors when they are experiencing an average degree of negative affectivity in response to a social stress compared to when they are too negatively affected by an emotional stress or even compared to when in an emotionally neutral state as when in the cognitive stress and control conditions. Moreover, after experiencing social stress the fact of being good emotion regulators promotes even more helping behaviors. In other words, it would make sense to expect grater donations, for example to charities, when people are either experiencing some distress but not too much or when they are very good at regulating their distress. At the same time, when individuals are in an extremely negative affective state due to emotional distress, they are much less willing to donate. This data should give a heads up to organizations relaying on charity donations in times when the population is experiencing major distress, just like is happening now during the worldwide COVID-19 pandemic.

In a time when people of all socio-economic backgrounds struggle due to either emotional distress due to COVID-19 and restrictive measures, cognitive challenges related to on line working while juggling family and house cores and social stress due to lack of social contacts for long periods of time and subsequently the return to social gatherings and interactions this study gives important indication on whether giving behaviors should be expected in relation to different distressing situations. Moreover, the slow reopening after the immunization following vaccine administration and gradual return to normality might be associated to the experience of social stress. Indeed, people might be overwhelmed by going back to daily and possibly judging social interactions. This source of stress, however, especially among better self-regulators may promote willingness to help and might be a significant period to ask for donations.

## Data Availability Statement

The datasets presented in this study can be found in online repositories. The names of the repository/repositories and accession number(s) can be found at: https://osf.io/pc5jt/?view_only=55392060b26d4d4cac9bddbe4202422f.

## Ethics Statement

The studies involving human participants were reviewed and approved by Ethical Committee for the Psychological Research of the University of Padova (Ethic Approval Number: 90898E5831387486DC98DB0C57111546). The patients/participants provided their written informed consent to participate in this study.

## Author Contributions

AH was responsible for the conceptualization and design, data collection and analysis, and original draft preparation and editing. LM contributed to designing, data collection, and writing. ER and SS supervised the conceptualization, data analysis, and writing. All authors contributed to the writing and approved the submitted version.

## Conflict of Interest

The authors declare that the research was conducted in the absence of any commercial or financial relationships that could be construed as a potential conflict of interest.

## Publisher’s Note

All claims expressed in this article are solely those of the authors and do not necessarily represent those of their affiliated organizations, or those of the publisher, the editors and the reviewers. Any product that may be evaluated in this article, or claim that may be made by its manufacturer, is not guaranteed or endorsed by the publisher.

## Footnotes

1 The present data have been collected during the 2020 pandemic of COVID-19, therefore this variable was accounted for in the manuscript both controlling for fear of COVID-19 and including families of COVID-19 patients as potential targets for donations.

2 To select the most adequate condition to be compared with a severe COVID-19 illness a pretest was conducted during the summer (July–August 2020). During the pretest, COVID-19 was compared with flu, pneumonia, and melanoma (given the relatively comparable prevalence and mortality rates of these illnesses in Italy) in terms of affective response to the illness, perceived danger, and probability of getting the illness and reported importance to help patients suffering because of the illness. Results showed that flu was the most comparable illness with the COVID-19.

3 The IAPS stimuli manipulated for the emotional stress were labeled with the following slide numbers; and the sequence was kept similar: 2141, 2095, 3030, 3530, 2703, 3053, 2800, 3080, 9940, 3170, 6300, 3140, 2799, 3160, 6230, 3213, 2683, 3215, 3059, 3185, 6212, 3101, 2780, 3102, 6210, 3195, 3230, 3005.1, 3103, 3001, 3261, 6250, 3216, 2205, 9910, 9911. While for the reminder the following stimuli were used: 6840, 3266, 9050, 9007, 9183, 9040, 9413, 3350, 9414, 6623, 9491, 9432, 9430, 3300, 9000, 9810.

4 In support to the conclusion that stress induction was not influenced by the pandemic, the same pattern of results was found when controlling for the fear of COVID-19 scale.
