# Supplementary figures and images for: Stress and Emotional Intelligence Shape Giving Behavior: Are There Different Effects of Social, Cognitive, and Emotional Stress?
Source: Front Psychol. 2022 Feb 24;13:800742. doi: 10.3389/fpsyg.2022.800742 (PMC8907929; doi:10.3389/fpsyg.2022.800742)

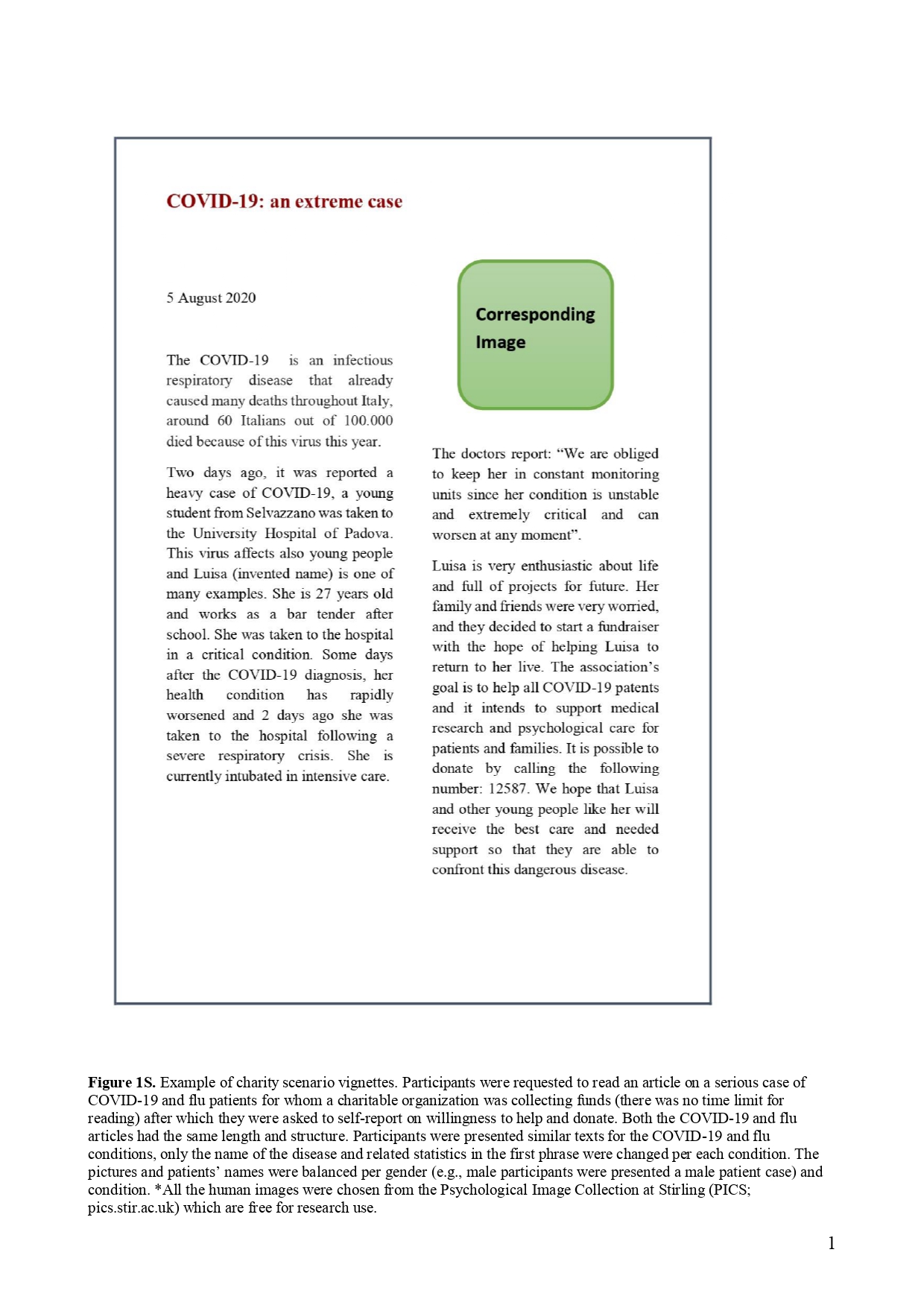

Supplement: Supplementary file 1 [file Image_1.jpg]
