# Supplementary material for: Stress and Emotional Intelligence Shape Giving Behavior: Are There Different Effects of Social, Cognitive, and Emotional Stress?
Source: Front Psychol. 2022 Feb 24;13:800742. doi: 10.3389/fpsyg.2022.800742 (PMC8907929; doi:10.3389/fpsyg.2022.800742)
Supplement: Supplementary file 2 [file Table_1.DOCX]

**Table 1S.** Descriptive statistics of changes of all negative affect states. Scores were computed as the difference between what participants have reported after each type of the four sources of stress and what had been reported before the stressor took place.

|  | **Cognitive Stress** | | **Emotional Stress** | | **Social Stress** | | **Control** | |
| --- | --- | --- | --- | --- | --- | --- | --- | --- |
|  | *M* | *SD* | *M* | *SD* | *M* | *SD* | *M* | *SD* |
| Afraid | -0.26 | 1.02 | 0.63 | 1.13 | 0.31 | 1.42 | -0.43 | 0.74 |
| Upset | 0.45 | 1.13 | 0.47 | 1.20 | 0.99 | 1.36 | -0.25 | 0.81 |
| Miserable | 0.40 | 0.81 | 0.86 | 1.19 | 0.10 | 1.22 | -0.22 | 0.73 |
| Depressed | -0.02 | 0.58 | 0.21 | 0.86 | 0.23 | 0.62 | -0.20 | 0.68 |
| Disgusted | 0.07 | 0.57 | 2.09 | 1.35 | 1.17 | 0.78 | -0.06 | 0.46 |
| Embarrassed | 0.93 | 1.18 | -0.48 | 1.07 | 2.19 | 1.37 | -0.08 | 0.97 |
| Furious | 0.02 | 0.81 | 0.60 | 1.24 | 0.09 | 0.86 | -0.07 | 0.49 |
| Guilty | 0.09 | 0.79 | 0.22 | 0.77 | -0.10 | 0.55 | -0.20 | 0.69 |
| Unhappy | -0.06 | 0.74 | 0.74 | 1.13 | -0.33 | 0.83 | -0.26 | 0.70 |
| Anxious | 0.55 | 1.23 | 0.11 | 1.07 | 0.68 | 1.32 | -0.21 | 1.03 |
| Scared | -.32 | 0.71 | 0.33 | 1.02 | -0.21 | 0.93 | -0.33 | 0.72 |
| Sad | -0.13 | 1.05 | 1.22 | 1.29 | -0.55 | 1.10 | -0.39 | 0.90 |
| Alone | -0.17 | 0.62 | 0.00 | 0.79 | -0.34 | 0.99 | -0.21 | 0.52 |
| Terrified | 0.07 | 0.67 | 0.58 | 1.06 | 0.32 | 1.08 | -0.33 | 0.72 |
| Shocked | 0.48 | 1.20 | 1.55 | 1.36 | 0.62 | 1.35 | -0.39 | 0.83 |
