# Supplementary material for: Stress and Emotional Intelligence Shape Giving Behavior: Are There Different Effects of Social, Cognitive, and Emotional Stress?
Source: Front Psychol. 2022 Feb 24;13:800742. doi: 10.3389/fpsyg.2022.800742 (PMC8907929; doi:10.3389/fpsyg.2022.800742)
Supplement: Supplementary file 3 [file Table_2.DOCX]

**Table 2S.** Group comparisons of main variables.

|  | Cognitive | | Control | | Emotional | | Social | |  |
| --- | --- | --- | --- | --- | --- | --- | --- | --- | --- |
|  | *N* | *M(SD)* | *N* | *M(SD)* | *N* | *M(SD)* | *N* | *M(SD)* | *t* |
| Changes in negative affect. | 101 | 2.13(6.03) | 95 | -3.64(5.65) | 91 | 9.12(9.22) | 96 | 3.62(7.67) | a: *t* = -6.91, *p* < .001  b: *t* = -11.44, *p* < .001  c: *t* = -7.45, *p* < .001  d: *t* = -6.28, *p* < .001  e: *t* = -1.53, *p* = .13  f: *t* = -4.44, *p* < .001 |
| Trait Emotional Intelligence | 100 | 5.02(0.65) | 94 | 4.92(0.84) | 90 | 5.02(0.70) | 96 | 5.00(0.65) | a: *t* = -.88, *p* = .38  b: *t* = -0.86, *p* = 0.39  c: *t* = -0.71, *p* = .48  d: *t* = -0.04, *p* = .97  e: *t* = 0.18, *p* = .86  f: *t* = -0.21, *p* = .83 |
| Fear related to COVID-19 | 101 | 24.82(8.82) | 95 | 25.64(9.91) | 91 | 25.39(8.61) | 96 | 25.74(8.33) | a: *t* = 0.61, *p* = .54  b: *t* = 0-18, *p* = .86  c: *t* = -0.07, *p* = .94  d: *t* = -0.45, *p* = .65  e: *t* = -0.74, *p* = .45  f: *t* = 0.28, *p* = .78 |
| Empathy | 101 | 62.02(15.42) | 95 | 65.00(7.24) | 91 | 64.00(9.57) | 96 | 62.06(13.21) | a: *t* = 1.71, *p* = .09  b: *t* = 0.80, *p* = .42  c: *t* = 1.90, *p* = .06  d: *t* = -1.05, *p* = .29  e: *t* = -0.02, *p* = .98  f: *t* = -1.14, *p* = .25 |

*Note:* a = Control vs Cognitive, b = Control vs Emotional, c = Control vs Social; d = Cognitive vs Emotional, e = Cognitive vs Social; f = Social vs Emotional
